# Case Report: Life-saving emergency chemotherapy in a critically ill patient with suspected metastatic testicular cancer prior to histological confirmation

**DOI:** 10.3389/fonc.2026.1901770

**Published:** 2026-07-29

**Authors:** Victoria John von Freyend, Moritz Kleemiss, Alexander Cox, Katjana Schwab, Peter Brossart, Michael Serries

**Affiliations:** 1 Department of Oncology, Hematology, Immuno-Oncology and Rheumatology, University Hospital Bonn, Bonn, Germany; 2 Center for Integrated Oncology (CIO) ABCD, Aachen Bonn Cologne Dusseldorf, Bonn, Germany; 3 Department of Urology and Pediatric Urology, University Hospital Bonn, Bonn, Germany

**Keywords:** acute respiratory failure, case report, critical care, emergency chemotherapy, metastatic germ cell tumor, oncological emergency

## Abstract

**Introduction:**

Patients with metastatic germ cell tumors undergo therapy including orchiectomy, chemotherapy and residual tumor resection. Even in case of metastatic disease, the treatment is still administered with curative intent. We present a case in which chemotherapy was successfully applied in an emergency setting prior to having histological confirmation of the suspected diagnosis of a germ cell tumor on our intensive care unit (ICU) while the patient was requiring mechanical ventilation, continuous renal replacement therapy (CRRT) and vasopressor support.

**Case presentation:**

A 35-year-old man presented with massive testicular swelling, severe dyspnea, bilateral leg edema and acute postrenal kidney failure. A computed tomography (CT) scan revealed extensive pulmonary and retroperitoneal metastases and pneumonia. He was admitted to our intensive care unit with multiorgan dysfunction requiring mechanical ventilation, CRRT and vasopressor support. Laboratory findings revealed moderately elevated beta-human chorionic gonadotropin (570 mIU/ml) and an elevated lactate dehydrogenase over 2000 U/L. Due to his critical condition and high clinical suspicion of a metastatic germ cell tumor, chemotherapy according to the PEI regimen (cisplatin, etoposide, ifosfamide) was initiated only one day after the admission to our ICU before histological confirmation of the diagnosis. The patient showed marked clinical, laboratory and radiological improvement after one cycle. An orchiectomy later revealed necrotic tissue with residual seminoma. Subsequent cycles required dose reductions due to prolonged kidney failure, infectious complications during aplasia and cardiomyopathy leading to heart failure. Application of ifosfamide was discontinued after the patient suffered from severe ifosfamide-related toxicities, including Fanconi-syndrome and neurotoxicity. A Positron Emission Tomography-CT scan after four cycles demonstrated an overall regression of the tumor with residual lesions.

**Conclusion:**

Advanced testicular cancer can present as an acute emergency. In selected life-threatening situations, immediate chemotherapy is justified in patients with high clinical suspicion of a germ cell tumor, even in the absence of histological confirmation. The combination of clinical findings, the pattern of metastases, morphological findings in imaging and tumor markers should be used to define the most likely diagnosis beforehand.

## Introduction

Young patients with metastatic germ cell tumors undergo therapy including orchiectomy, chemotherapy and residual tumor resection. Even in a case of metastatic disease, the treatment is still administered with curative intent (1–3). We present a case in which chemotherapy was successfully applied in an emergency setting prior to having histological confirmation of the suspected diagnosis of a germ cell tumor on our intensive care unit (ICU) while the patient was requiring mechanical ventilation, continuous renal replacement therapy (CRRT) and norepinephrine as vasopressor support.

In the majority of cases germ cell tumors are found in young men between 15 and 45 years of age with a good prognosis and a cure rate of approximately 95% including all tumor stages (1, 4). The most frequent first symptom is a non-painful swelling and hardening of the testicle. In advanced cases this is sometimes accompanied by weight loss or dyspnea. The further diagnostic procedures would be identifying potentially elevated tumor markers (human chorionic gonadotropin (beta-hCG), alpha-fetoprotein (AFP) and lactate dehydrogenase (LDH)), performing an ultrasound of the testicles, a computed tomography (CT) scan of the thorax, the abdomen and the pelvis and in defined cases also a Magnetic Resonance imaging (MRI) of the head. The next step would be to perform an orchiectomy or at least obtain a biopsy of one of the metastases (5, 6). Based on the findings, the appropriate chemotherapy regimen is chosen.

## Case presentation

**Figure 1:**
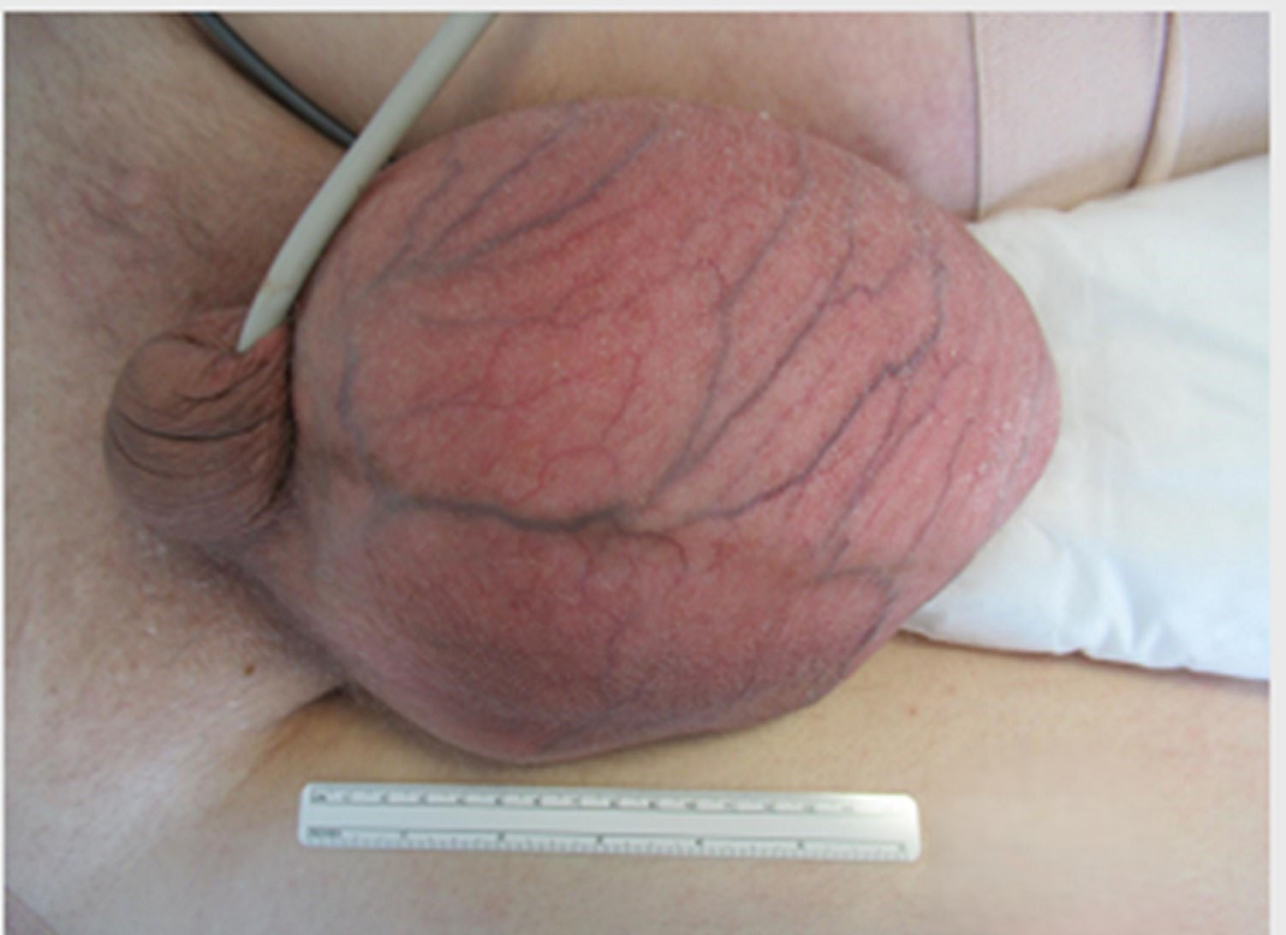
Large swelling of the testicles one day after admission to our ICU (circumference 48cm, length 20cm).

**Figure 2:**
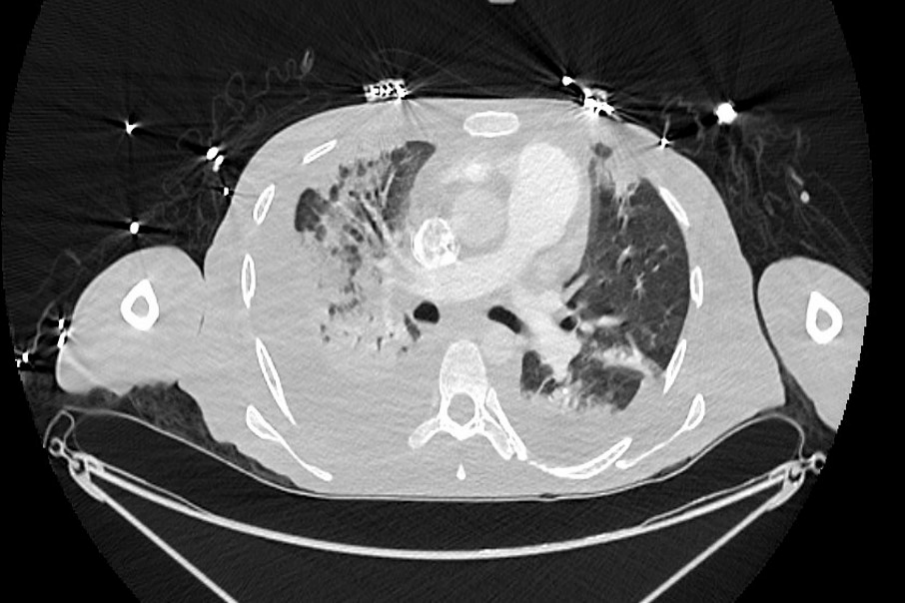
Initial CT scan of the thorax following endotracheal intubation showing extensive pulmonary metastases and pneumonia.

We present the case of a 35-year-old man, the father of two sons, who initially presented at another hospital with severe swelling of the left testicle as shown in **Figure 1** (circumference 48 centimeters (cm)), severe dyspnea, and bilateral leg edema. According to the patient`s history, the swelling of the testicle began approximately six months prior to the hospital admission and he had already lost over 15 kilograms of his bodyweight. The laboratory findings showed an elevated D-dimer and kidney failure. An ultrasound revealed severe congestion of both kidneys, an extensive deep vein thrombosis involving four venous levels in the left leg and a big tumor in the left testicle. CT imaging demonstrated pulmonary metastases and pneumonia as shown in **Figure 2**, without evidence of pulmonary embolism. Furthermore, the CT scan revealed retroperitoneal and parailiac tumorous formations. A double-J catheter was inserted into both ureters; on the left side, an additional percutaneous nephrostomy had to be placed in the upper part of the kidney to ensure adequate drainage. During the procedure, the patient experienced respiratory failure and required endotracheal intubation due to septic shock from pneumonia and widespread pulmonary metastases. At that time, the patient was referred to our hospital for further evaluation of a suspected metastatic germ cell tumor.

Due to advanced postrenal renal failure, CRRT was initiated. Furthermore, the patient`s respiratory status deteriorated requiring high ventilatory pressures (PEEP 8 mbar, Peak Inspiratory Pressure 28 mbar) due to pneumonia and extensive pulmonary metastases. It was not possible to implement lung protective ventilation settings. Moreover, deep vein thrombosis greatly increased the risk of pulmonary embolism. Tumor markers showed moderate elevation of beta-hCG (570 mIU/ml), LDH over 2000 U/L and normal AFP. These findings were indicative of a seminoma, although the beta-hCG was expected to be higher given the tumor burden. Beta-hCG is not a highly sensitive tumor marker, being elevated in approximately 20–30% of seminoma cases and about 50% of non-seminomatous germ cell tumors. However, due to its high specificity, an elevated β-hCG level is strongly indicative of an underlying germ cell tumor (7). Given the patient’s young age and occasional testicular involvement, lymphoma was the leading differential diagnosis (8–10). At this point in time, we would typically try to obtain a biopsy of the tumor before starting chemotherapy. However, the patient’s condition was acutely life-threatening, with three organs already failing, making an orchiectomy too risky and leaving no time to await pathology results or delay treatment by even a few days. For this reason, we decided to give chemotherapy according to the PEI regimen (cisplatin 20 mg/m^2^ day 1-5, etoposide 75 mg/m^2^ day 1-5, ifosfamide 1200 mg/m^2^ day 1-5) based on our leading diagnosis, a metastatic germ cell tumor. Bleomycin was contraindicated due to respiratory failure. Primary prophylaxis with granulocyte-colony stimulating factor was applied alongside PEI protocol. Brain MRI is recommended in patients with extensive pulmonary metastases, however, the duration of the examination was not tolerable for the patient at that time (6). A cranial CT performed shortly after the first cycle of chemotherapy showed no evidence of brain metastases. Following the first cycle of chemotherapy, the patient`s respiratory status improved and we tried to extubate the patient one week after intubation. Although the patient was young and one would usually expect a sufficient level of physical fitness, his respiratory muscles proved too weak to cough up secretions independently due to cancer cachexia, which made reintubation necessary. Following an additional failed extubation, the patient underwent a tracheostomy. The CRRT was discontinued after approximately two weeks. Finally, after the first cycle of therapy and following the time of aplasia, the orchiectomy was performed. The histopathologic finding was mostly necrosis with occasional tumor cells which were characterized as most likely seminoma cells. Ultimately, the diagnosis of metastatic seminoma was confirmed and classified as intermediate prognosis according to the IGCCCG criteria. The beta-hCG level in the patient`s blood had already decreased to 21 mIU/ml after one cycle of chemotherapy. Because of the below explained prolonged kidney failure and further complications we had to reduce the dosage of chemotherapy during the second cycle (etoposide and ifosfamide each reduced to 75% of the initial dose) and switch cisplatin to carboplatin. During the third and fourth cycle ifosfamide was no longer applied due to severe toxicities.

**Figure 3:**
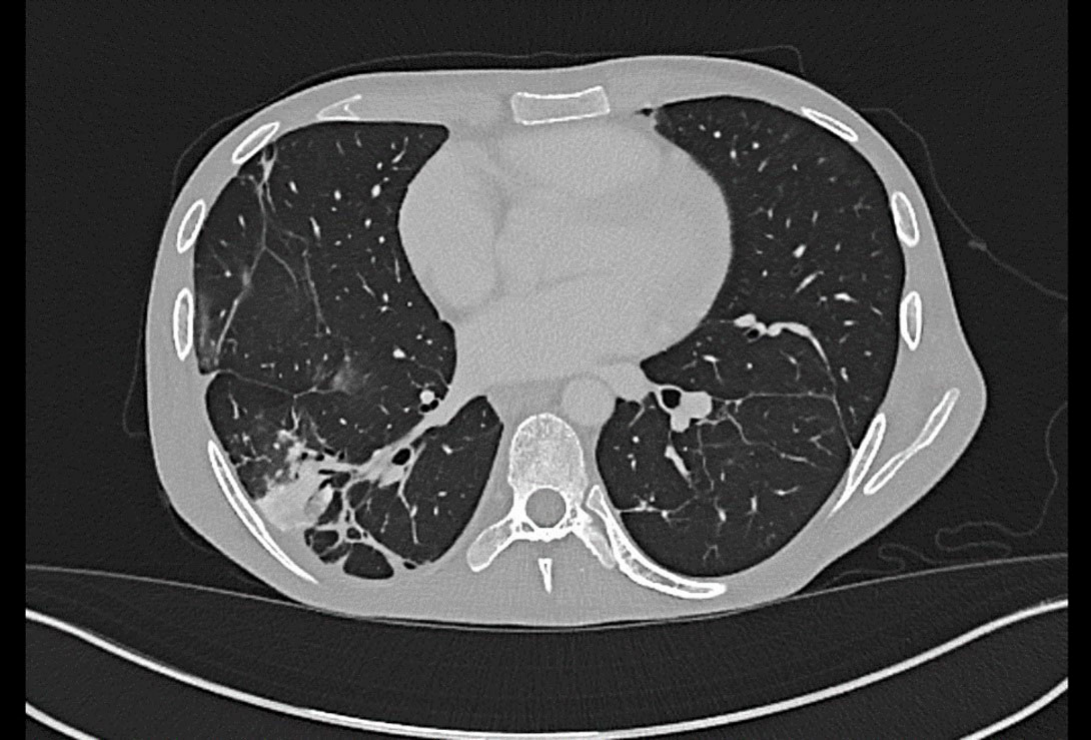
CT scan of the thorax after two cycles of chemotherapy showing regression of metastases.

A CT scan after two cycles of chemotherapy already showed improvement of the pulmonary metastases as shown in **Figure 3**.

## Complications

Weaning was prolonged due to the extensive pulmonary metastases, the patient`s delayed presentation to the hospital and already weakened respiratory muscles and cancer cachexia. Decannulation of the tracheostomy tube was achieved only after a month and a half in our ICU. Furthermore, the patient suffered several toxicities caused by chemotherapy. After having recovered from postrenal kidney failure the patient developed Fanconi-Syndrome induced by ifosfamide which is a disorder of the proximal renal tubule preventing reabsorption of vital substances including glucose, phosphate, electrolytes, bicarbonate, and amino acids. This leads to excessive urinary losses, metabolic acidosis and various biochemical abnormalities (11). We treated Fanconi-Syndrome by substitution of bicarbonate and electrolytes and intravenous fluids to compensate the polyuria. He also suffered from neurotoxicity caused by Ifosfamide with a single seizure and temporary disorientation and reduced vigilance which resolved within a couple of days. Due to insufficient evidence, we did not use methylene blue to treat ifosfamide induced neurotoxicity (12).

In times of aplasia following the first two cycles the patient developed neutropenic fever with candidemia (caused by candida tropicalis) and HSV-mucositis after the first cycle and sepsis due to Enterobacter cloacae bacteremia following the second cycle. After the third cycle he suffered another episode of neutropenic fever.

Moreover, an echocardiography showed heart failure with reduced ejection fraction after the second chemotherapy cycle (Ejection fraction of 36%) with MRI showing cardiomyopathy. Possible causes for cardiomyopathy were prolonged uremia, infectious complication during aplasia or another toxicity caused by Ifosfamide. Cardiomyopathy was managed with guideline-directed heart failure therapy with dose adjustments necessitated by hypotension and renal impairment. Finally, he suffered prolonged cytopenia with a thrombocyte count below 30 G/l over eight weeks following the second cycle of chemotherapy, therefore we had to delay the application of the third cycle. A bone marrow biopsy showed toxic bone marrow damage.

## Follow-up and outcome

**Figure 4:**
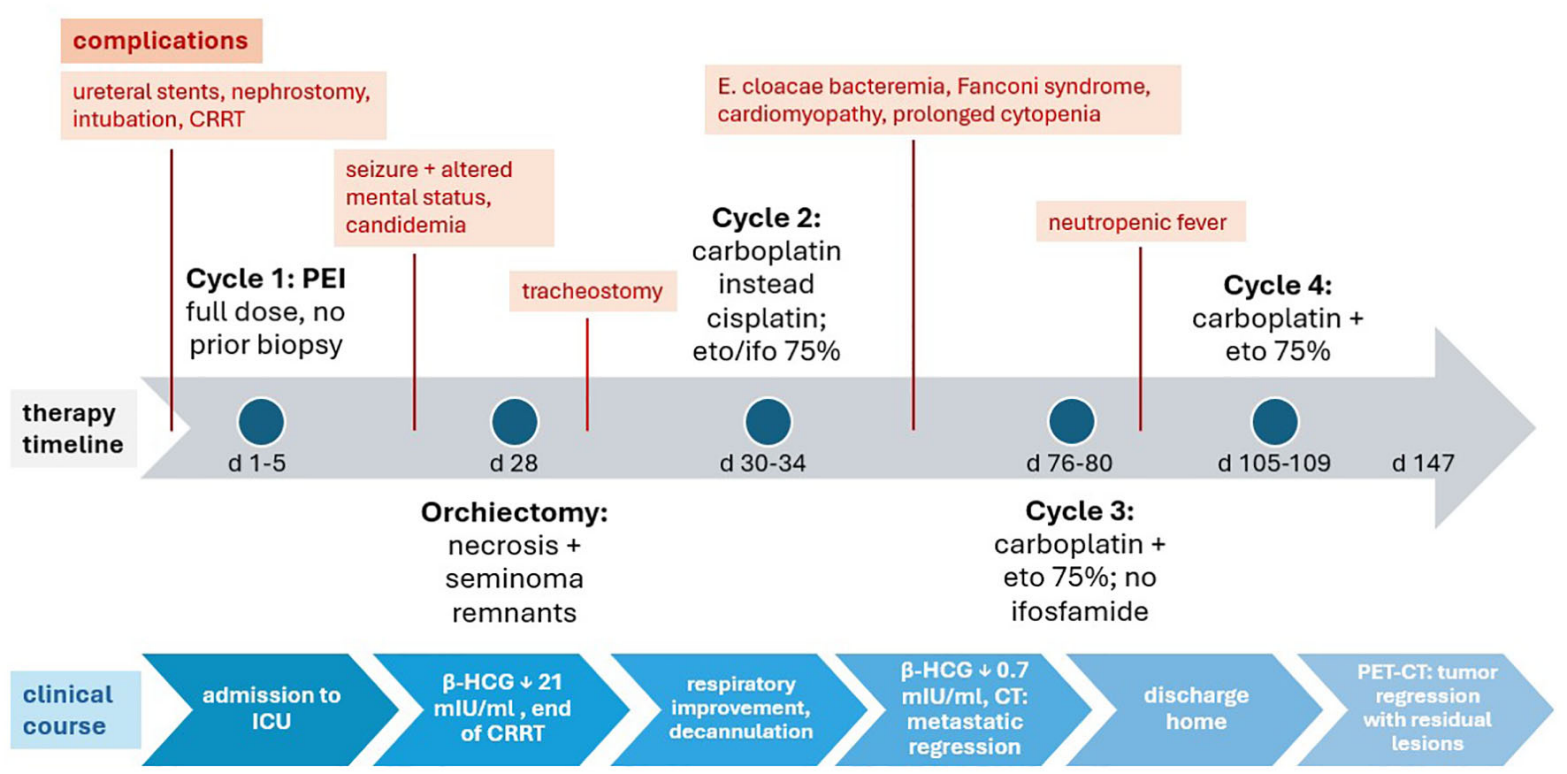
Timeline of the patient including therapeutic steps (black), clinical course (blue) and complications (red). d, day; PEI, cisplatin/etoposide/ifosfamide; eto, etoposide; ifo, ifosfamide; 75%, reduction to 75% of the initial dose.

**Figure 4** provides an overview of the patient’s clinical course. The patient received four cycles of chemotherapy. Between the third and fourth cycle the patient was discharged. He required additional support from his family and assistive devices such as a rollator, a nursing care bed and a bedside commode. Ongoing urinary drainage is required through both a DJ stent and the left-sided nephrostomy catheter. The previously elevated tumor markers have normalized. After four cycles a fluorodeoxyglucose (FDG)- positron emission tomography (PET)/CT scan was performed. It demonstrated an overall regression of the tumor mass with residual lesions in the retroperitoneal lymph nodes and the lung with moderate to intense tracer uptake. The total residual tumor burden measured over 3 cm. Given normalized tumor markers and morphological residual disease, a follow-up FDG PET/CT was indicated 6 weeks after the prior study, as up to 14% of scans may yield false-positive results attributable to post-chemotherapy inflammatory reactions (13, 14). Depending on the findings, histologic confirmation by biopsy will be pursued to verify viable tumor tissue, most likely followed by salvage chemotherapy.

## Discussion

This case illustrates a case of life-threatening cancer in a young patient and improvement through prompt introduction of chemotherapy one day after admission to the intensive care unit. Usually, chemotherapy is not a typical lifesaving measure in the ICU but in this case the metastatic tumor was the cause of already two organs failing. The question is whether it is justified to give chemotherapy by clinical und laboratory findings prior to confirming the suspected diagnosis histologically. In clinical literature, there is little available evidence relating to such highly acute germ cell tumor cases, which underlines the importance of sharing this experience. Critical considerations supporting the decision to proceed without awaiting pathological results include the acute life-threatening condition and the patient`s extreme clinical fragility, which raised concern that he might not have survived surgery.

Additionally, pulmonary or retroperitoneal metastases are difficult to access for biopsy in patients on mechanical ventilation. Furthermore, the pattern of metastasis, the moderately elevated beta-HCG, the ultrasound and clinical findings of the testicle strongly indicate the presence of a germ cell tumor (6). Approximately 95 percent of malignant tumors arising in the testis are germ-cell tumors (2). However, lymphoma in the testicles should be considered as the most probable differential diagnosis (7–9). In our case the patterns of metastases and elevation of beta-hCG made the presence of a lymphoma highly unlikely. Nevertheless, tumor markers such as beta-hCG and AFP may be considered relatively specific indicators for germ cell tumors but they may also be present in other forms of cancer. In the literature, case reports have described patient`s with gastric cancer, ovarian cancer or anal cell squamous carcinoma with elevated beta-hCG (15, 16). Therefore, tumor markers should be interpreted within the broader clinical context rather than be used as a stand-alone diagnostic tool. Another differential diagnosis to consider was choriocarcinoma, however this is an extremely rare diagnosis and these tumors usually produce high levels of beta-hCG. In summary, regarding the patient`s young age, the testicular mass with ultrasound criteria for malignancy, the metastatic pattern and the elevated tumor markers, the diagnosis of a germ cell tumor, in particular seminoma, remained the most likely diagnosis (7).

An important consideration is that the patient was suffering from an acute infection (pneumonia) which could have worsened when undergoing chemotherapy. Additionally, we did not have a confirmed diagnosis and could potentially cause harm with choosing an inappropriate chemotherapy. Following one cycle of chemotherapy there was a significant risk that a histological diagnosis could not be obtained as the tumor had been largely destroyed and only necrotic tissue remained. This was shown in multiple studies where an orchiectomy was performed after chemotherapy because the initial biopsy was obtained from one of the metastases (5, 17, 18).

Another case report described a young patient with suspected germ cell tumor and metastases involving the lungs and the left atrium, resulting in severe dyspnea. In that case, surgical resection of the cardiac metastases was performed before the initiation of chemotherapy to reduce the risk of tumor embolism, stroke, and pulmonary venous obstruction (19). In contrast, the patient’s critical clinical condition in our case precluded surgical intervention. Moreover, the patient’s dyspnea was attributable not only to extensive pulmonary metastatic disease but also to concomitant pneumonia. Complex cases such as the one presented here require an individualized treatment approach and should be discussed within a multidisciplinary tumor conference to determine the optimal treatment strategy.

Because of the multiple complications by the administered chemotherapy, it has to be discussed whether any dose reduction should have been made in the first place or whether because of the critical clinical condition of the patient on admission the toxicities were unavoidable. Chemotherapy according to the PEI regimen is the recommended standard treatment for patients with metastatic seminoma and an intermediate prognosis, as defined by German and European clinical practice guidelines, in case bleomycin is contraindicated (6, 20). A possible option would have been to replace cisplatin with carboplatin initially due to the acute kidney injury (20). In a direct comparison between carboplatin-based chemotherapy (bleomycin, etoposide, carboplatin) and bleomycin, etoposide and cisplatin (BEP) in metastatic germ cell tumors, the combination with carboplatin was inferior to BEP (21). Because of the critical condition our patient was in due to the tumor we decided to use a powerful full dose regimen under CRRT. The choice of chemotherapy regimen had to be made only within 24 hours because of the patient´s rapid clinical deterioration. Furthermore, the renal failure was attributed to tumor compression, which could only be resolved by rapid tumor shrinkage. CRRT was not discontinued during chemotherapy administration in order to prevent drug accumulation and fluid overload caused by the intravenous solutions. Considering the patient´s young age and generally favorable prognosis of metastatic germ cell tumors even in metastatic disease, treatment was pursued with curative intent (1, 2) in retrospect, given the significant toxicities the patient experienced, it must be considered whether dose reductions especially in the first cycle might have achieved sufficient tumor reduction while improving tolerability. However, due to the urgency of the situation, a full-dose regimen was chosen to reduce tumor burden as quickly as possible. The decline of beta-hCG levels in the blood after only one cycle acted as a surrogate parameter for chemotherapy response (22).

In the German Guideline for germ cell tumors (2020) there is an expert consensus on that in life-threatening situation with an impending respiratory failure, high tumor burden and high tumor markers, especially beta-hCG, one can consider giving chemotherapy without histologic confirmation beforehand. In our case the beta-hCG was only moderately elevated, but we still predicted the right diagnosis.

## Conclusion

Advanced testicular cancer can present as an acute emergency. In selected life-threatening situations, immediate chemotherapy may be justified in patients with high clinical suspicion of a germ cell tumor, even in the absence of histological confirmation. The combination of clinical findings (such as testicular swelling), the pattern of metastases, morphological findings in imaging and tumor markers should be used to define the most likely diagnosis beforehand.

Early treatment can be lifesaving even in patients with multiorgan failure. However, histological confirmation following chemotherapy may not always be feasible, so this therapeutic approach should be reserved judiciously for select critical cases.

## Limitations

Here, we provide a single example in which this approach was successful and proved lifesaving for the patient. More experience and comparable cases are needed to prove the reliability of our conclusions.

## Data Availability

The original contributions presented in the study are included in the article/**Supplementary Material**. Further inquiries can be directed to the corresponding author.
